# The Development of a Practical Artificial Intelligence Tool for Diagnosing and Evaluating Autism Spectrum Disorder: Multicenter Study

**DOI:** 10.2196/15767

**Published:** 2020-05-08

**Authors:** Tao Chen, Ye Chen, Mengxue Yuan, Mark Gerstein, Tingyu Li, Huiying Liang, Tanya Froehlich, Long Lu

**Affiliations:** 1 School of Information Management Wuhan University Wuhan China; 2 School of Information Technology Shangqiu Normal University Shangqiu China; 3 Division of Biomedical Informatics Cincinnati Children’s Hospital Medical Center Cincinnati, OH United States; 4 Department of Pediatrics University of Cincinnati College of Medicine Cincinnati, OH United States; 5 Program in Neurodevelopment and Regeneration Yale University New Haven, CT United States; 6 Department of Molecular Biophysics and Biochemistry Yale University New Haven, CT United States; 7 Program in Computational Biology and Bioinformatics Yale University New Haven, CT United States; 8 Department of Computer Science Yale University New Haven, CT United States; 9 Children Nutrition Research Center Chongqing China; 10 Children's Hospital of Chongqing Medical University Chongqing China; 11 Ministry of Education Key Laboratory of Child Development and Disorders Chongqing China; 12 China International Science and Technology Cooperation Base of Child Development and Critical Disorders Chongqing China; 13 Chongqing Key Laboratory of Translational Medical Research in Cognitive Development and Learning and Memory Disorders Chongqing China; 14 Guangzhou Women and Children's Medical Center Guangzhou China; 15 Guangzhou Medical University Guangzhou China; 16 Division of Developmental and Behavioral Pediatrics Cincinnati Children’s Hospital Medical Center Cincinnati, OH United States

**Keywords:** autism spectrum disorder, magnetic resonance imaging, neuroimaging, brain, histogram of oriented gradients, cluster analysis, classification, machine learning

## Abstract

**Background:**

Autism spectrum disorder (ASD) is a complex neurodevelopmental disorder with an unknown etiology. Early diagnosis and intervention are key to improving outcomes for patients with ASD. Structural magnetic resonance imaging (sMRI) has been widely used in clinics to facilitate the diagnosis of brain diseases such as brain tumors. However, sMRI is less frequently used to investigate neurological and psychiatric disorders, such as ASD, owing to the subtle, if any, anatomical changes of the brain.

**Objective:**

This study aimed to investigate the possibility of identifying structural patterns in the brain of patients with ASD as potential biomarkers in the diagnosis and evaluation of ASD in clinics.

**Methods:**

We developed a novel 2-level histogram-based morphometry (HBM) classification framework in which an algorithm based on a 3D version of the histogram of oriented gradients (HOG) was used to extract features from sMRI data. We applied this framework to distinguish patients with ASD from healthy controls using 4 datasets from the second edition of the Autism Brain Imaging Data Exchange, including the ETH Zürich (ETH), NYU Langone Medical Center: Sample 1, Oregon Health and Science University, and Stanford University (SU) sites. We used a stratified 10-fold cross-validation method to evaluate the model performance, and we applied the Naive Bayes approach to identify the predictive ASD-related brain regions based on classification contributions of each HOG feature.

**Results:**

On the basis of the 3D HOG feature extraction method, our proposed HBM framework achieved an area under the curve (AUC) of >0.75 in each dataset, with the highest AUC of 0.849 in the ETH site. We compared the 3D HOG algorithm with the original 2D HOG algorithm, which showed an accuracy improvement of >4% in each dataset, with the highest improvement of 14% (6/42) in the SU site. A comparison of the 3D HOG algorithm with the scale-invariant feature transform algorithm showed an AUC improvement of >18% in each dataset. Furthermore, we identified ASD-related brain regions based on the sMRI images. Some of these regions (eg, frontal gyrus, temporal gyrus, cingulate gyrus, postcentral gyrus, precuneus, caudate, and hippocampus) are known to be implicated in ASD in prior neuroimaging literature. We also identified less well-known regions that may play unrecognized roles in ASD and be worth further investigation.

**Conclusions:**

Our research suggested that it is possible to identify neuroimaging biomarkers that can distinguish patients with ASD from healthy controls based on the more cost-effective sMRI images of the brain. We also demonstrated the potential of applying data-driven artificial intelligence technology in the clinical setting of neurological and psychiatric disorders, which usually harbor subtle anatomical changes in the brain that are often invisible to the human eye.

## Introduction

### Background

Autism spectrum disorder (ASD) is a heterogeneous disorder characterized by social impairments, communicative deficits, and restricted, repetitive behaviors. According to the 2018 Centers for Disease Control and Prevention report on autism, approximately 1% (1/59) of US children aged 8 years have been diagnosed with ASD, which represents an increase compared with previous reports [1]. The diagnosis and intervention costs of ASD are growing in concert with the increasing prevalence. A recent study predicted that treatment costs will rise to US $461 billion in 2025 if the prevalence rate of ASD holds steady at present rates and that costs will rise to US $1 trillion by 2025 if the prevalence rate of ASD continues to steeply rise as seen over the last decade [1]. However, concerns have been raised about the accuracy and validity of the reported increase in ASD prevalence, as many other neurobehavioral conditions, as well as variations in developmentally normal behaviors, share common features with ASD and may be misdiagnosed as ASD [2]. Inappropriate ASD diagnoses, and therefore potentially inappropriate applications of ASD-related therapies, stand to increase economic burden. Conversely, deferred or missed ASD diagnosis in children meeting the diagnostic criteria, which appears to be a particular problem for certain sociodemographic [3] and clinical groups [4], lead to a delay in receipt of services and place children at risk for worse outcomes. Therefore, appropriate and early ASD diagnosis and intervention is of crucial importance to improve prognostic outcomes and reduce economic costs.

ASD is now diagnosed mainly by clinical behavior-based approaches, which incorporate standardized tools such as the Autism Diagnostic Observation Scale and Autism Diagnostic Interview-Revised scale. However, this approach is subjective and time consuming [5]. Although it has been reported that ASD has a strong genetic basis, genetic markers are not currently used in the diagnostic process as ASD etiology is complex and the full complement of autism-associated genes is unclear. As magnetic resonance imaging (MRI) is a widely used noninvasive examination method to detect brain abnormalities in clinical practice, there is much interest in its potential to improve or refine the ASD diagnostic process. In clinics, structural MRI (sMRI) has been successfully used to facilitate the diagnosis or treatment of space-occupying lesions such as tumors [6,7]. However, the structural changes of the brain in neurological and psychiatric disorders are not as salient as tumors; thus, it is difficult for clinicians to discover the subtle anatomical changes in the brain. Many studies have focused on finding the functional connectivity abnormalities in the brain using functional MRI (fMRI). Indeed, investigators have explored the use of fMRI to identify ASD. For example, Guo et al [8] developed a deep neural network model using the functional connectivities between brain regions based on the resting-state fMRI. Price et al [9] combined dynamic functional connectivity features in a multinetwork algorithm to classify childhood autism. Huang et al [10] fused multiple functional connectivity networks for ASD diagnosis. However, although fMRI can image cerebral hemodynamics with high spatial resolution, the high cost may limit its potential as a widely used ASD diagnostic tool in clinics [11]. More importantly, it is difficult to interpret the functional connectivity-based results owing to the impact of the underlying brain structure, cognitive state, and subject motion during data acquisition [12]. Furthermore, a recent study suggested that the statistical software used to analyze the raw data from fMRIs might be significantly flawed [13].

Compared with fMRI, sMRI has less data requirements, is more commonly used in clinical settings, and is more amenable to populations for whom compliance is a challenge as it can be performed under sedation. Many ASD sMRI studies have used morphometric features, such as brain surface area, volume, and thickness, to distinguish ASD from control images [14,15]. For example, a recent study of infants at high risk for ASD found hyperexpansion of the cortical surface area and expanded brain volumes in those later diagnosed with ASD [16]. In addition, some studies have made strides toward elucidating ASD brain morphology. Specifically, Bigler et al [17] observed differences in the frontal lobe, parietal lobe, temporal lobe, limbic system, and cerebellum structures for patients with ASD versus healthy controls.

### Related Work

Although sMRI images can provide brain anatomical change information, errors in interpretation can occur owing to difficulty in verifying these subtle changes solely by visual examination. In addition, as there is abundant genetic, phenotypic, and clinical heterogeneity among individuals with ASD, these morphometric features alone are insufficient for diagnosing ASD in clinical settings given that each individual feature is unlikely to be present in the full range of individuals meeting the ASD criteria. To address such barriers, in recent years, machine learning algorithms have been developed to identify underlying brain change patterns in other neurobehavioral conditions marked by similar degrees of heterogeneity. When applying machine learning algorithms to sMRI data, image features representing the sMRI image need to be extracted first. Some of these features are adapted from traditional morphology approaches, while others are developed specifically for machine learning approaches. The traditional morphometric features can be classified into region of interest (ROI), voxel-based morphometry (VBM) [18], surface-based morphometry (SBM) [19], deformation-based morphometry (DBM) [20], and tensor-based morphometry (TBM) [21,22]. Unfortunately, the ROI, VBM, SBM, DBM, and TBM approaches all have significant limitations. Owing to requiring manual or semimanual delineation of brain regions, the ROI process may be labor intensive and time consuming [23]. The performance of VBM, DBM, and TBM methods is highly sensitive to registration accuracy, which is difficult to achieve [24], and is reliant on deformation registration, which may cause over-alignment problems [25]. The SBM method is unable to admit subcortical structures, such as the amygdala and basal ganglia, which may play crucial roles in ASD [26]. To address the limitation of traditional image features discussed earlier, local image features developed specifically for machine learning approaches, such as scale-invariant feature transform (SIFT) [27], do not depend on precise deformation registration. SIFT is assumed to be invariant to image translation, scaling, and rotation and robust to local geometric distortion, which has already been applied to analyze brain images [25,28-31]. However, SIFT itself has several shortcomings. Although SIFT can improve classification accuracy compared with traditional morphometry features, it uses an expert-designed approach to identify visually salient changes that may not relate to the disease. Moreover, SIFT can only describe the characteristics of a limited number of key points and the regions around the key points. However, given that abnormal brain regions in neurodevelopmental disorders/diseases may occur in any position and may be very small, they may be overlooked by the SIFT modality.

Given the above limitations in traditional image features as well as SIFT, another prominent local image feature called histogram of oriented gradients (HOG) [32] has been widely used in computer vision applications (eg, human detection [33,34], vehicle classification [35,36], traffic sign detection [37], pose estimation [38], and general image classification [39]). As HOG can describe the distribution of intensity gradients or edge directions well, it is useful for characterizing local object appearance and shape [32]. In addition, as HOG features can filter most of the nonessential information (eg, a constant colored background) while providing an output of multiple bidimensional histograms for a brain region to reflect the changes within a brain region, HOG features are good at reflecting small or subtle anomalies that may be ignored by SIFT. In prior studies, HOG has generally been used to describe 2D images. Although 2D HOG can be applied to a 3D image, the 3D image needs to be sliced into a series of 2D images along a certain orientation, which can be problematic as changes induced by the disease may be evident only at specific orientations. Fortunately, a recently developed modality called 3D HOG can be analyzed directly inside the 3D volumetric image, which allows image gradient information for the abnormal region to be kept in a more discriminative 3D form and therefore improves classification performance.

### Objectives

To address the unique challenges inherent in the neuroimaging studies of ASD, we therefore proposed a novel 2-level classification framework called histogram-based morphometry (HBM), which is based on the 3D HOG feature extraction method. Instead of processing the whole brain image, we divided the entire brain into a few local regions with a given size, which is the foundation of our 2-level hierarchical framework. The first-level classifier is designed for the local regions related to diseased or healthy status, while the second-level classifier or final classifier is for the entire brain that is represented with the concatenation of each region’s status. The 3D HOG is computed not for the entire brain but for each local brain region. By using the HBM classification framework, we can classify individuals as patients with ASD or healthy controls. Moreover, the classification contribution of each local HOG feature can be calculated and those features contributing most to the disease classification result can be used to distinguish the predictive brain regions associated with ASD.

This paper has presented the development of the 3D HOG and HBM methods, as well as their application to ASD datasets. In the Methods section, we have described the data source, data preprocessing, 3D HOG feature design, 2-level HBM framework development, and the experimental design. In the Results section, we have discussed the experiment results derived from the analysis of data from the second edition of the Autism Brain Imaging Data Exchange (ABIDE II) [40]. We have concluded by contextualizing our results and discussing the outlook for future ASD neuroimaging research.

## Methods

### Data Acquisition and Preprocessing

In this study, we used sMRI data from ABIDE II, which includes 19 datasets collected at 18 sites (2 datasets were collected at the same site) and 1114 subjects (521 patients with ASD and 593 healthy controls). For each subject, the ABIDE II datasets consist of resting-state fMRI images, T1-weighted sMRI images, and phenotypic information. Some sites also include diffusion tensor imaging data that may be used to investigate the structural abnormalities of white matter. As an enhancement to the first edition of the Autism Brain Imaging Data Exchange (ABIDE I) datasets, ABIDE II provides greater phenotypic characterization than ABIDE I data to better address the 2 key sources of heterogeneity: psychiatric co-occurring illness and female sample percentage [40]. The inclusion and diagnostic criteria for patients with ASD and healthy controls are different between each site, and details of the criteria are described in the study by Martino et al [40]. From the 17 datasets, we chose 4 datasets collected from 4 sites, including ETH Zürich (ETH), NYU Langone Medical Center: Sample 1 (NYU), Oregon Health and Science University (OHSU), and Stanford University (SU). Data from a total of 119 patients with ASD and 131 healthy controls from across these 4 sites were used for these analyses. Table 1 lists the sample overview for each site. Age is an important factor that may affect different characteristics, for example, cortical thickness, of the brain in ASD. To evaluate the applicability of our proposed HBM method to different age ranges, we chose the 4 datasets that represent distinct age distributions among all the datasets. Specifically, to reduce the impact of multisite data heterogeneity, we first used single-site data for model classification performance evaluation. Then, we combined all the data from the 4 datasets to evaluate model capability to deal with data heterogeneity.

As the ABIDE II data are original Digital Imaging and Communications in Medicine (DICOM) images, in the first step of data preprocessing, we used the MRIcron tool to convert DICOM images to NifTI images. Then, data processing was performed using SPM12 (UCL Queen Square Institute of Neurology, United Kingdom), which is a third-party package for MATLAB (MathWorks, Natick, Massachusetts, United States). All converted structural images were segmented and normalized to an Montreal Neurological Institute (MNI) standard space.

**Table 1:** Overview of participants in the 4 training datasets.

Index	Dataset	ASD^a^, n (male/female)	Healthy controls, n (male/female)	Age (years), mean (SD)	Age range (years)
1	ETH^b^	13 (13/0)	24 (24/0)	22.7 (4.4)	14-31
2	NYU^c^	48 (43/5)	30 (28/2)	9.8 (4.9)	5.2-34.8
3	OHSU^d^	37 (30/7)	56 (27/29)	10.9 (2.0)	7-15
4	SU^e^	21 (19/2)	21 (19/2)	11.1 (1.2)	8-13
5	Mixed^f^	119 (105/14)	131 (98/33)	12.4 (5.6)	5.2-34.8

^a^ASD: autism spectrum disorder.

^b^ETH: ETH Zürich.

^c^NYU: NYU Langone Medical Center: Sample 1.

^d^OHSU: Oregon Health and Science University.

^e^SU: Stanford University.

^f^Mixed: dataset combining data from all the 4 datasets.

### Developing the 3D Histogram of Oriented Gradients Feature

**Figure 1:**
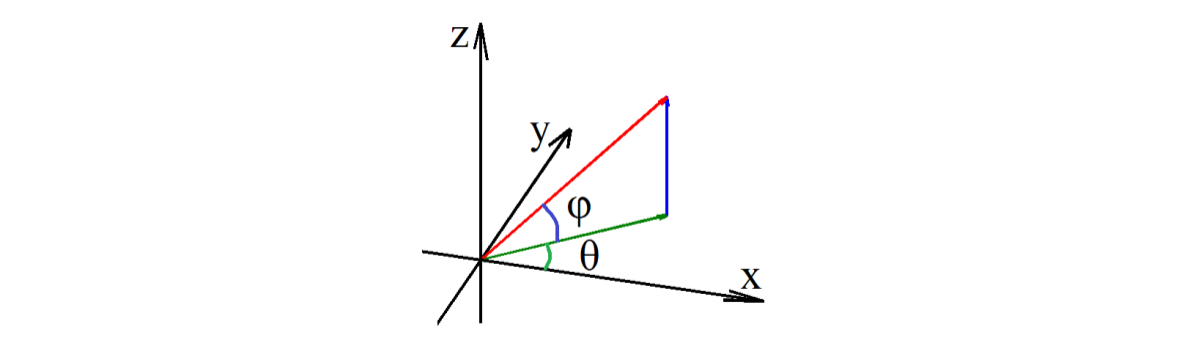
Two angles related to gradient direction calculation in 3D space.

In the process of extending the concept of HOG from a 2D space to 3D space, we needed to define the methods for calculating the image gradient (including direction and magnitude) and partitioning the gradient directions into a few orientation bins (or channels) in a 3D space. The gradient directions in the 3D space were represented by using 2 angles, theta and phi, as shown in Figure 1. Then, the gradient of each image voxel is calculated based on these 2 angles (see Multimedia Appendix 1 for more details).

Similar to 2D HOG, the gradient direction in 3D HOG also needed to be partitioned into several orientation bins. The difference lies in that the partitions in 2D HOG are spread over 360° in just one 2D plane, while the partitions in 3D HOG are spread over the entire volumetric space. There are many partition schemes to divide the orientation space. We have introduced the 2 partition schemes as follows.

**Figure 2:**
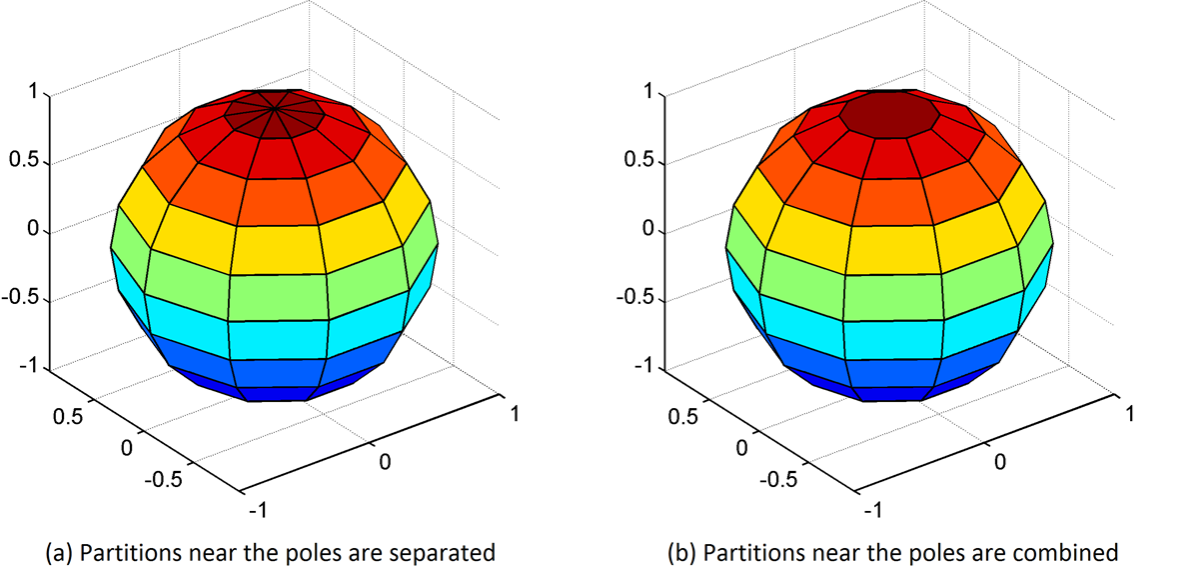
Two partition schemes of the orientation bins in 3D space.

The first scheme is to allocate the orientation bins in horizontal and vertical directions with equal-space angle ranges, such as the 2D HOG, and each bounded area between the 2 directions is considered as one 3D partition. The partition results are shown in Figure 2.

When every partition area is projected onto the sphere surface, they correspond to the surface area between the latitude and longitude lines. For this partition scheme, the number of orientation bins, which is equal to the dimension number of the 3D HOG features, is calculated using the following equation in: 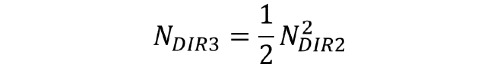 where *N*_DIR3_ is the number of directions in 3D space and N_DIR2_ is the number of directions in 2D space.

In Figure 2, part (a), for the partitions near the poles, a slight change in the angles will result in a different orientation bin assignment. This causes the features to be overly sensitive to the angle differences in some but not all directions. To avoid potential performance loss because of this phenomenon, we proposed an additional partition scheme, in which the partitions adjacent to the pole points are combined into 1 partition as shown in Figure 2, part (b).

The number of orientation bins for this second partition scheme, which merges the direction areas near the pole into 1 direction, is calculated using the equation in:


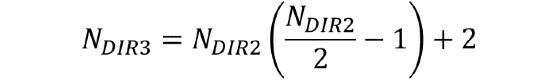


For the convenience of calculation, the value of *N*_DIR2_ is constrained to be an even number. For example, if *N*_DIR2_ is set to 8, *N*_DIR3_ will be 32 as calculated in the first scheme while in the second scheme *N*_DIR3_ will be 26.

### Overall Classification Framework

**Figure 3:**
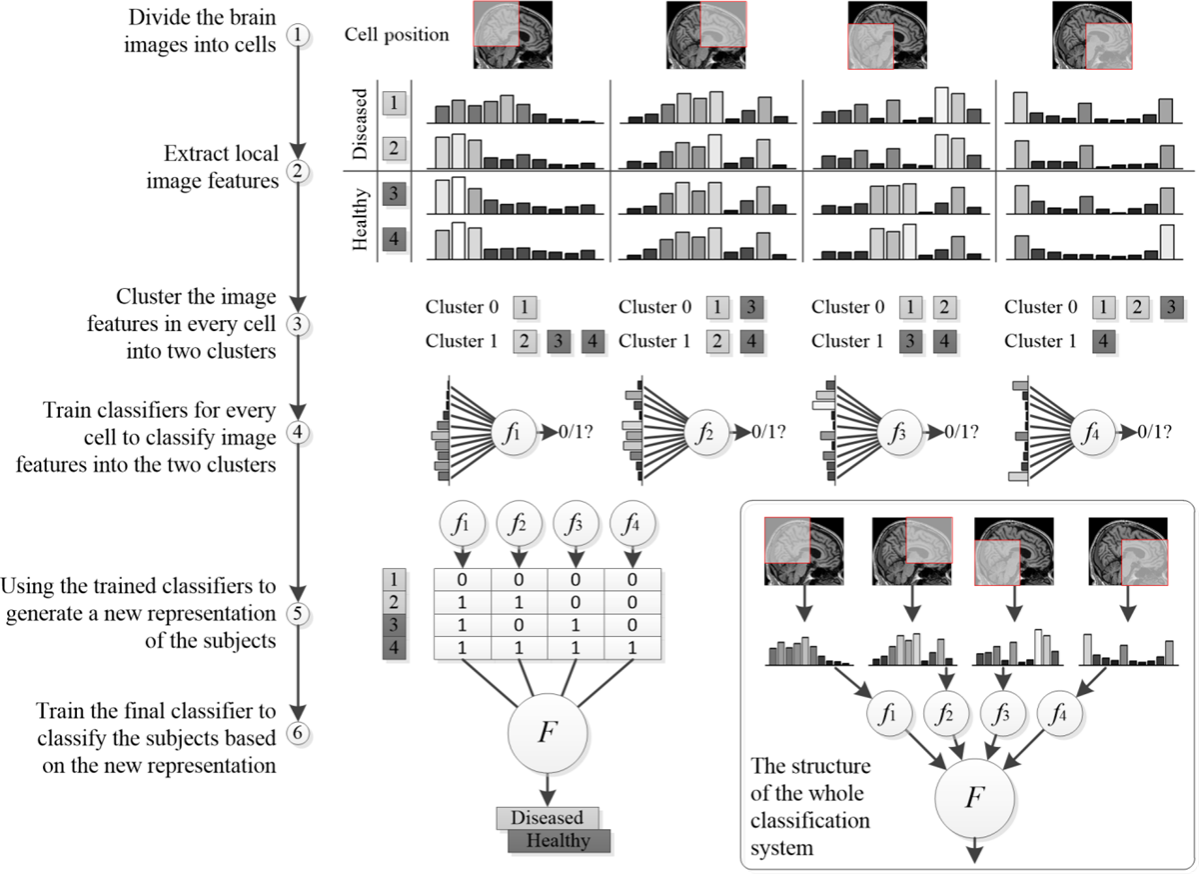
Overview of the proposed histogram-based morphometry (HBM) classification framework.

In this paper, we proposed a 2-level HBM classification framework based on 3D HOG features to differentiate between patients with ASD and healthy controls. Each brain image was firstly divided into a densely overlapping grid of regional cells, and the 3D HOG feature of each cell was computed. On the basis of the brain division, we developed a first-level classification algorithm to predict whether a given cell provides strong evidence to support a final disease/health classification. As there is no label for each cell, a clustering algorithm was used to first find the labels for each cell (the details have been discussed in the following sections). Then, a second-level classification was used to make a final classification based on all the evidence from each cell. Figure 3 shows the 2-level classification framework using a 2D image example for convenient illustration. The bottom-right part of the figure represents the testing process, while the remaining part shows the training process.

### Algorithm Steps

#### Brain Image Division and Local Feature Extraction

Before the feature extraction step, we first divided the entire 3D MRI brain image into regional cells in step 1. This brain division method can be applied not only to 3D MRI volumetric images, in which a regional cell equates to a *cube*, but to 2D MRI slices, in which a regional cell equates to a *square*. In our algorithm, we computed the HOG feature for each cell but did not collect it into a combined feature vector used to represent the entire image. In the standard HOG usage, all the local HOG features were combined into a high-dimensional feature vector used as input to the classifier [32]. In our hierarchical classification framework, these local features were transformed into high-level forms that can reduce the dimensionality of the features input to the final classifier, which has the benefit of reducing overfitting in the relatively small-sized datasets that are often available in medical studies. Furthermore, using local features is helpful to identify the ASD-related brain regions that have large feature contributions to the disease classification result. In image division, cell size and cell overlapping percentage are 2 important parameters that will affect the classification accuracy. Therefore, different brain image division schemes should be evaluated to determine which has the best classification performance.

In step 2, we extracted local HOG features using 2 different gradient direction partition schemes: HOG-32 and HOG-26, as shown in parts (a) and (b) in Figure 2, respectively. A comparison between these 2 schemes is also necessary to determine which has superior performance. Of note, better classification performance using the 3D HOG algorithm usually results from MRI scans with high spatial resolution, while the performance of the 3D HOG algorithm may degrade if the MRI scan has a low spatial resolution. In this case, an alternative 2D HOG algorithm may be used.

#### Local Feature Clustering and Regional Classifier Training

In step 3, we worked on each cell independently. For each cell, the goal was to find a binary representation to indicate whether it is related to the diseased status or healthy status. However, we did not have a class label for each cell. Although the class label of the whole brain is known in training samples, it does not mean that each cell should have the same class label as the whole brain. Even in a diseased subject, there may be a lot of cells in the brain that look perfectly normal. Owing to the unknown class label for each cell, we applied a clustering algorithm to the training samples to get the class labels of individual cells. As the distribution of clusters is unknown, we tried 2 different clustering algorithms, such as K-means and hierarchical clustering, that are suitable for different cluster distributions. Although the clustering algorithm works well during the training stage, we proposed to use a classification algorithm to generate the binary representation during the testing stage. The reason we used classification instead of clustering during the testing stage was because we did not need to keep all the training features while using the approach to make a prediction, which makes the method more scalable and practical. Thus, based on the clustering labels of cells in training samples, we built regional classifiers in step 4 for predicting the cell status of test samples. When the K-means algorithm is used for clustering, the resulting clusters usually have a spherical shape in feature space and the centroids are good exemplars for the corresponding clusters. Therefore, the nearest centroid classification method was used in this case. If the hierarchical algorithm is used for clustering, the centroids of the clusters may not be representative of the cluster, and therefore, the nearest centroid classifier is not appropriate. In this case, the support vector machine (SVM) can be used to build regional classifiers for testing samples.

#### Compact Feature Representation and Final Classifier Training

The labeled local features only reflect the status of brain regions and not the whole picture of the characteristics of the brain. Therefore, in step 5, we concatenated each local feature status of 1 brain image into a new high-level compact feature representation of that image. For model training, we constructed the high-level feature by directly concatenating the clustering results obtained in step 4. Of note, the clustering result of each feature was concatenated according to a certain sequence, for example, from top-left to bottom-right on the grid. Such a sequence is actually determined by the HOG feature extraction algorithm, and the same sequence is also used when concatenating the binary status of HOG features, thus ensuring the unified meaning of feature representation for all samples. On the basis of the new feature representation and diagnosis labels of the training data, we trained the final classifier using the SVM classification method in step 6. SVM is one of the most widely used classifiers that can perform not only linear classification but also nonlinear classification [41]. It has already been applied to various diseases and neurodevelopmental disorders, for example, Parkinson disease [42], Alzheimer disease [43,44], ASD [45,46], attention-deficit/hyperactivity disorder [47], and schizophrenia [48].

#### Process for the Test Sample Classification

The abovementioned steps describe the whole training process of obtaining the 2-level classification models including the regional classifier and the final classifier. We could then apply these classifiers to unknown test samples. First, 3D local HOG features of the cells in a test brain image are extracted with the same method as the training process. Then the regional classifiers, such as the nearest centroid, are used to classify each local HOG feature into disease-related or healthy-related labels. These labels are then concatenated to generate the compact representation of that test image. Finally, the final classifier is applied to predict whether the test sample is a patient with ASD using the compact feature vector as the input to the classification model.

### Feature Contribution Calculation

Besides using the HBM framework to make a classification of the test sample, we could also investigate each cell’s feature contribution to the algorithm’s prediction that each participant is a patient with ASD versus a healthy control. A higher value of the feature contribution indicates more likelihood of a cell being disease-related. As we used the SVM method in the final classification level, the feature contribution could be calculated based on the coefficients of the linear SVM classifier. However, this method can cause problems as we do not know which clustered label represents the diseased status. Thus, we chose the Naive Bayes approach instead to calculate the feature contribution for both clustered labels. In the strictest sense, the *feature contribution* calculated by the Naive Bayes method should be called *feature importance*, which only reflects the feature contribution given that the final classifier is a Naive Bayes classifier. We will explore more interpretable mapping from the local features to the final classification results in future research.

First, we will introduce the Naive Bayes approach, which is based on Bayes’ theorem. This approach has been widely used for classification in many domains owing to its simplicity and strong performance. It is assumed that predictive features *X*_0_, *X*_1_, …, *X*_n_ are independent of each other given the state of a class variable *Y*. Although it is difficult to reduce the dependence for a neuroimage analysis because different brain regions are correlated in many ways by nature, empirical observations have suggested that the Naive Bayes works quite well even when there is dependence between features [49]. Therefore, we used Bayes’ theorem to derive the posterior probability *P* (*Y* | *X*_0_, *X*_1_, …, *X*_n_) as follows:


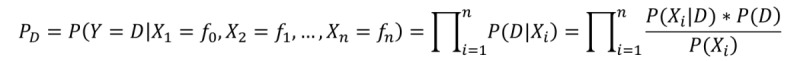



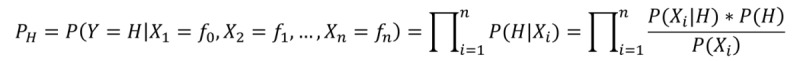


where *X*_i_ ∈{0, 1} represents the *i*th cell clustering result, and *Y* ∈ {*D*, *H*} represents the training sample label. In addition, *P*_D_ and *P*_H_ refer to the probability of being classified as a patient with ASD versus a healthy control, respectively, conditioned on the state of each cell. If *P*_D_ > *P*_H_, we predicted that the test sample is more likely to be a patient with ASD than a healthy control. To avoid underflow in the Bayesian computation, we used the log ratio as follows:


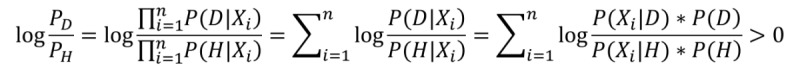


where we defined the log sum item 
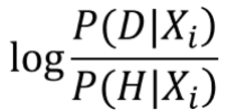
 as the feature contribution at the *i*th cell. A higher value of this item indicates a more predictive feature. It is worth noting that because we did not know exactly which cell state (0 or 1) indicates a disease-related feature and these 2 feature states can both contribute to the disease, we calculated both of their feature contributions.

Then, according to the first-level classification results of each cell in a test patient sample, the most predictive features whose contribution values are above a preset threshold can be identified. We set a threshold on the feature contribution to just show the top features to the patients (in a hypothetic clinical use case). The threshold is usually set to different values when using heterogeneous sMRI data from different sites or when the parameter values (eg, cell size and cell overlapping percentage) are changed. The cells that contribute most to the classification result of ASD are considered to be the candidate regions related to the disease.

### Experimental Design

In the 2-level HBM framework, we evaluated the 2 different 3D gradient direction partition schemes using the algorithm combinations for feature clustering, regional classifier training, and final classifier training listed in Table 2. The performance of the 4 instances listed in the table will be compared later. The instance name in the table (eg, KNS32) is the abbreviation created using the first letter from the local feature clustering algorithm name (K-means), the regional classification algorithm name (nearest centroid), the final classification algorithm name (SVM), and 32 orientation bins.

**Figure 4:**
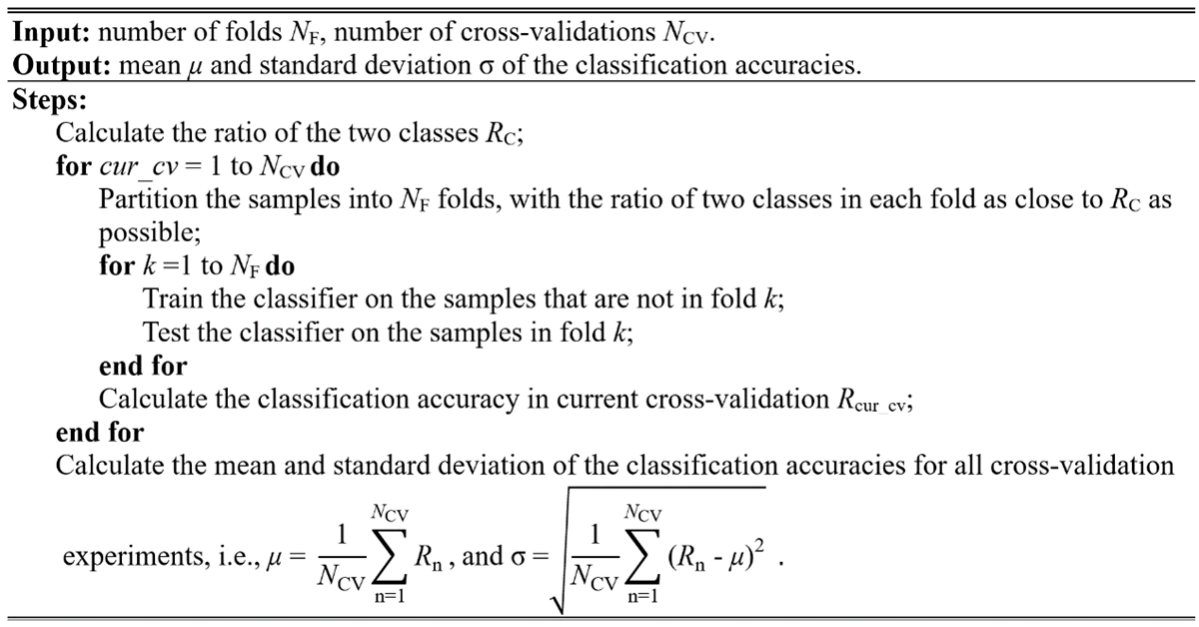
Algorithm of the stratified cross-validation with multiple random runs.

After the final classification model is trained, its performance is evaluated, typically via the cross-validation (CV) method. The widely used CV methods in brain image analysis include leave-1-out CV [25,48,50], leave-2-out CV [45,51,52], k-fold CV [53,54], and stratified k-fold CV [55,56]. Although there are conflicting reports in the literature, most papers, including a review of brain image classification methods, suggest that 10-fold CV is the most appropriate method [57]. In this study, we trained our model using the stratified 10-fold CV method. The stratified CV method provides the following advantages. First, the stratified method can keep the ratio of 2 sample classes in each fold as close to that of all samples as possible, retaining the original data distribution pattern of the entire dataset. Second, the variance of model performance estimations will decrease by performing several random runs, in each of which all samples are first shuffled and then split into a pair of training and test sets. The stratified CV method proposed in this paper is implemented as the pseudo-code shown in Figure 4.

In the 3D HOG partition scheme, there is a parameter *N*_DIR2_ that represents the number of orientation bins in either the horizontal or vertical direction of the 3D space. If *N*_DIR2_ is set too high, the computation speed of the algorithm will be slowed. However, more importantly, the feature will be more sensitive to noise and other noninformative signals in the images. Furthermore, the dimension of the feature will be high, which usually requires more samples to avoid the *curse of dimensionality*. Otherwise, if N_DIR2_ is set too low, details of the image will be lost. In this paper, we set the number of *N*_DIR2_ to the frequently used value 8, and the total number of directions in 3D space was 32 and 26 for the two 3D HOG partition schemes. The other parameters for the HOG features, including cell size and overlapping percentage, were evaluated using the CV method. The performance measures we used to evaluate our algorithm included classification accuracy, sensitivity, specificity, positive predictive value, negative predictive value, F1 score, and the area under the curve (AUC).

**Table 2:** The 4 instances of the proposed histogram-based morphometry framework used for performance evaluation.

Instance name	Image feature	Image feature processing for each cell		Final classification
		Clustering	Classification	
KNS32	HOG^a^-32^b^	K-means	Nearest centroid	SVM^c,d^
KNS26	HOG-26^e^	K-means	Nearest centroid	SVM^c^
HSS32	HOG-32^b^	Hierarchical	Linear kernel SVM	SVM^c^
HSS26	HOG-26^e^	Hierarchical	Linear kernel SVM	SVM^c^

^a^HOG: histogram of oriented gradients.

^b^HOG-32 is the histogram of oriented gradients feature with 8 directions in a 2D plane and 32 directions in 3D space.

^c^Three different kernels have been tested, for example, the linear kernel, the polynomial kernel, and radial base function kernel.

^d^SVM: support vector machine.

^e^HOG-26 is the HOG feature with 8 directions in a 2D plane, and the 2 poles are considered as 2 directions in 3D space; therefore, the total number of directions is 26.

## Results

### Comparing the Classification Performance of Different Histogram-Based Morphometry Instances

To compare the performance of the 4 HBM instances listed in Table 2, we used the stratified 10-fold CV evaluation method to obtain each performance measure. As the size of cell and the overlapping between 2 cells may influence the model’s performance, we performed a parameter scan for the best values of these 2 parameters. The cell size ranged from 10 voxels to 20 voxels and cell overlapping percentage ranged from 20% to 50%. In the final classification step, we tested 3 different SVM kernels, including the linear kernel, the polynomial kernel, and radial base function kernel. We then chose the linear kernel for use owing to its superior performance.

**Figure 5:**
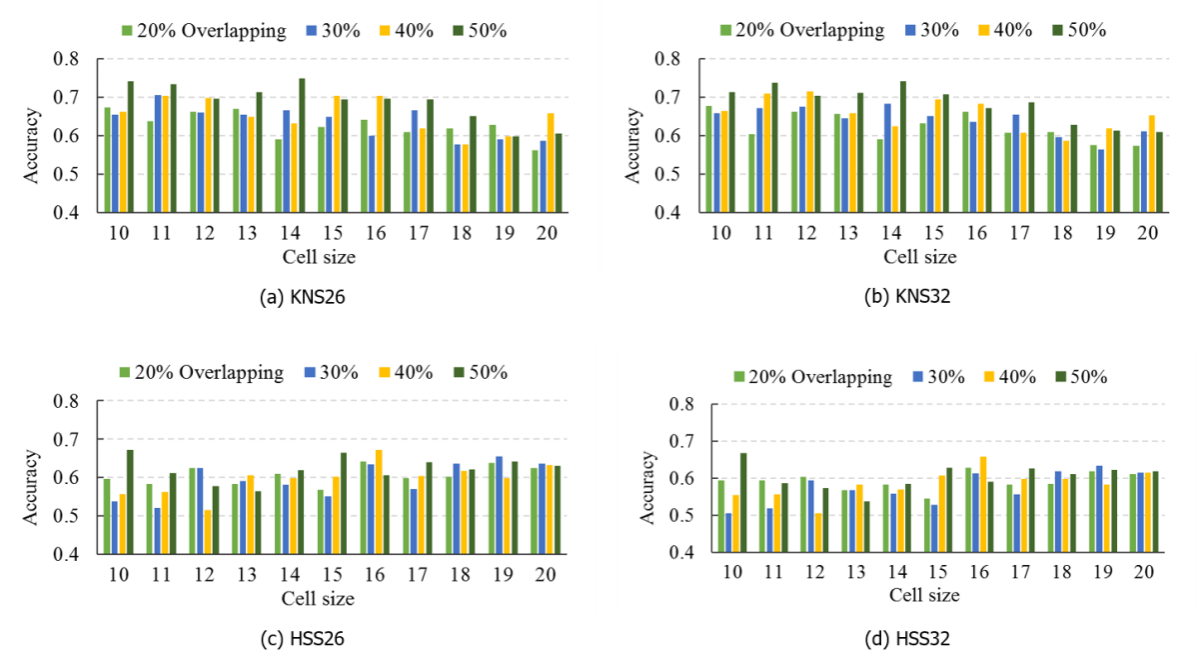
Classification accuracies for the NYU Langone Medical Center: Sample 1 dataset using 4 histogram-based morphometry (HBM) instances including KNS26 (a), KNS32 (b), HSS26 (c), and HSS32 (d).

Figure 5 shows the stratified 10-fold CV average accuracies based on the data from the NYU site when using different HBM instances and different parameter values. The expanded form of the abbreviations of the HBM instances in Figure 5 can be found in Table 2. From the figure, it can be seen that although the classification accuracies fluctuate as the parameter values change, KNS26 and KNS32 performed significantly better than HSS26 and HSS32, which means that the combination of K-means and centroid algorithms is more suitable for our proposed HBM framework. Meanwhile, Figure 5 shows that KNS26 outperformed KNS32 and HSS26 outperformed HSS32, which supports the rationality and effectiveness of the HOG-26 partition scheme. In addition, among the different parameter values, KNS26 obtained the best average classification accuracy, 74% (58/78), when the cell size was set to 14 voxels and the cell overlapping percentage was set to 50%. For the other 3 sites, ETH, OHSU, and SU, KNS26 also outperformed KNS32, although the best parameter values may be different (see Multimedia Appendix 2 for the results of these additional analyses). Of note, our method was not overly sensitive to the parameters, so model performance was generally good for a wide range of parameters.

### Comparing the Classification Performance of Different Local Feature Extraction Algorithms

In this paper, we used the HOG algorithm for local image feature extraction in the HBM framework. This algorithm helps to generate high-quality representations that depict image edge and texture. To evaluate the effects of different local feature extraction algorithms on classification performance, we also used SIFT, another widely used local feature detection algorithm, to extract features from brain images and developed an SVM approach to analyze the extracted SIFT features. This approach has been applied to neurological diseases such as Alzheimer disease [25,31], Parkinson disease [31], and bipolar disease [31]. As shown in Figure 5, KNS26 was the best performing HBM instance, so we compared it (rather than KNS32) with the SIFT-based SVM approach.

We trained both classifiers using the stratified 10-fold CV, and the training data were the same for them in each fold. The results show that a HOG-based KNS26 HBM approach achieves much better performance than the SIFT-based SVM approach (Tables 3 and 4). Overall, comparison results depicted in Tables 3 and 4 demonstrate that HOG features are more suitable for delineation of the underlying structural change patterns in sMRI images than SIFT features. By transforming the low-level HOG features into high-level features, our proposed 2-level HBM classification framework can effectively employ the high-level features to differentiate individuals as either patients with ASD or healthy controls. In the last row of Table 3, we can see that the performance degraded when building the model on data from the 4 datasets. We have discussed the reason in the Discussion section.

**Table 3:** Classification performance using histogram-based morphometry on the second edition of the Autism Brain Imaging Data Exchange datasets.

Dataset	Best parameter		Histogram-based morphometry (KNS26)											
	Cell size	Overlapping (%)	ACC^a^		SEN^b^		SPE^c^		PPV^d^		NPV^e^		F1^f^	AUC^g^
			N	n (%)	N	n (%)	N	n (%)	N	n (%)	N	n (%)		
ETH^h^	10	20	37	32 (86)	13	10 (77)	24	22 (92)	12	10 (83)	25	22 (88)	0.790	0.849
NYU^i^	14	50	78	58 (74)	48	40 (83)	30	18 (60)	52	40 (77)	26	18 (69)	0.805	0.787
OHSU^j^	19	40	93	70 (75)	37	23 (62)	56	46 (82)	33	23 (70)	60	46 (77)	0.662	0.794
SU^k^	17	20	42	30 (71)	21	17 (81)	21	13 (62)	25	17 (68)	17	13 (77)	0.751	0.763
Mixed^l^	12	30	250	162 (65)	119	87 (73)	131	76 (58)	142	87 (61)	108	76 (70)	0.662	0.650

^a^ACC: accuracy is the ratio of correctly classified subjects over all subjects.

^b^SEN: sensitivity is the ratio of correctly classified subjects with autism spectrum disorder (ASD) over all subjects with ASD.

^c^SPE: specificity is the ratio of correctly classified subjects without ASD over all subjects without ASD.

^d^PPV: positive predictive value is the ratio of correctly classified subjects with ASD over all predicted subjects with ASD.

^e^NPV: negative predictive value is the ratio of correctly classified subjects without ASD over all predicted subjects without ASD.

^f^F1: F1 score.

^g^AUC: area under the curve.

^h^ETH: ETH Zürich.

^i^NYU: NYU Langone Medical Center: Sample 1.

^j^OHSU: Oregon Health and Science University.

^k^SU: Stanford University.

^l^Mixed: dataset combining data from all the 4 datasets.

**Table 4:** Classification performance using scale-invariant feature transform and support vector machine on the second edition of the Autism Brain Imaging Data Exchange datasets.

Dataset	Performance using scale-invariant feature transform and support vector machine											
	ACC^a^		SEN^b^		SPE^c^		PPV^d^		NPV^e^		F1^f^	AUC^g^
	N	n (%)	N	n (%)	N	n (%)	N	n (%)	N	n (%)		
ETH^h^	37	24 (65)	13	8 (62%)	24	16 (67)	16	8 (50)	21	16 (76)	0.533	0.709
NYU^i^	78	44 (56)	48	29 (60)	30	15 (50)	44	29 (66)	34	15 (44)	0.624	0.595
OHSU^j^	93	52 (56)	37	19 (51)	56	33 (59)	42	19 (45)	51	33 (65)	0.482	0.605
SU^k^	42	18 (43)	21	10 (48)	21	8 (38)	23	10 (44)	19	8 (42)	0.449	0.367

^a^ACC: accuracy is the ratio of correctly classified subjects over all subjects.

^b^SEN: sensitivity is the ratio of correctly classified subjects with autism spectrum disorder (ASD) over all subjects with ASD.

^c^SPE: specificity is the ratio of correctly classified subjects without ASD over all subjects without ASD.

^d^PPV: positive predictive value is the ratio of correctly classified subjects with ASD over all predicted subjects with ASD.

^e^NPV: negative predictive value is the ratio of correctly classified subjects without ASD over all predicted subjects without ASD.

^f^F1: F1 score.

^g^AUC: area under the curve.

^h^ETH: ETH Zürich.

^i^NYU: NYU Langone Medical Center: Sample 1.

^j^OHSU: Oregon Health and Science University.

^k^SU: Stanford University.

### Comparing 3D Histogram of Oriented Gradients and 2D Histogram of Oriented Gradients

HOG features represent image edge and texture, and the feature quality is affected by MRI acquisition parameters, especially spatial resolution that is decided by slice thickness, matrix size, and field of view. Low spatial resolution will decrease image sharpness and cause fuzzy edges, which may degrade the classification performance. By contrast, high spatial resolution helps to retain more fine-grained and high-contrast information of the brain tissues, which enable us to extract HOG features directly in its inherent 3D form. From the anatomical scan parameters, we can see that the T1-weighted sMRI images are all high-resolution images in these 4 datasets. In our proposed 3D HOG algorithm, the features were extracted directly inside the 3D volumetric image. In the 2D HOG algorithm, the features were extracted from the 2D MRI slices. The hypothesis is that the 3D HOG algorithm will generate highly discriminative representations with higher quality than those generated by the 2D HOG algorithm.

**Figure 6:**
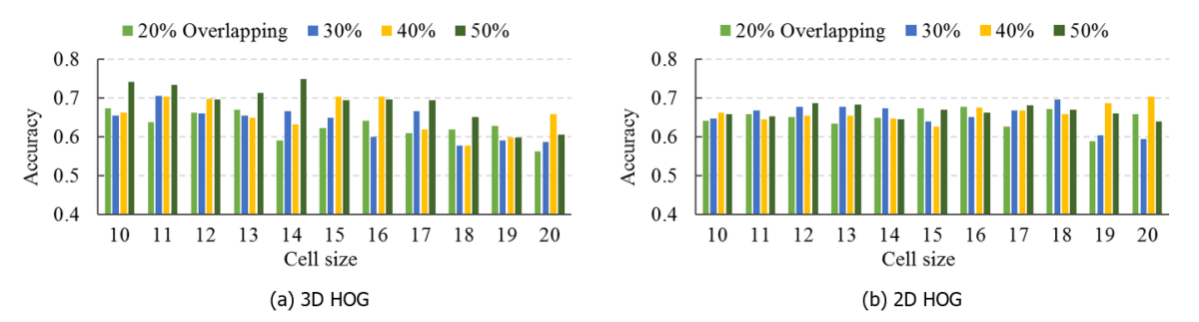
Classification accuracies for the NYU Langone Medical Center: Sample 1 dataset using a 3D histogram of oriented gradients (HOG; a) and 2D HOG (b).

To validate the hypothesis, we tested all the HBM instances listed in Table 2 for the 4 datasets. Here, data from the NYU site and KNS26 instance are used as examples to compare 3D HOG with 2D HOG. The evaluation scheme for both algorithms was the 10-fold CV, and the same parameter scan scope was used as discussed in the Comparing the Classification Performance of Different Histogram-Based Morphometry Instances section. Figure 6 presents the classification accuracy obtained from 3D HOG and 2D HOG. We can see from the figures that 3D HOG outperforms 2D HOG for some scan parameters and obtains the highest accuracy when the cell size is set at 14 voxels and cell overlapping percentage is set at 50%. The other 3 sites show a comparison result similar to NYU (see Multimedia Appendix 2 for the results of these additional analyses). Thus, the comparison between these 2 HOG algorithms supports the hypothesis that 3D HOG can generate more competitive representations for the ASD diagnosis task.

### Identifying Predictive Autism Spectrum Disorder–Related Brain Regions

**Figure 7:**
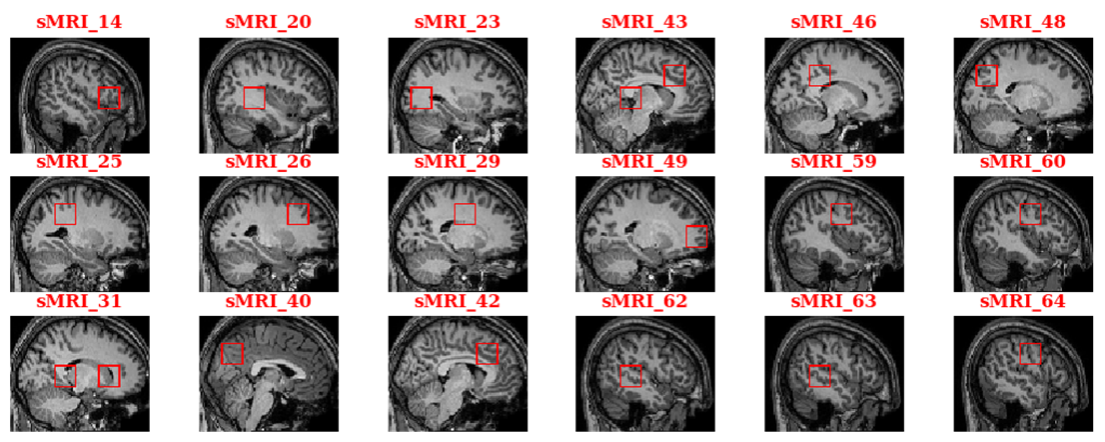
Annotation of the autism spectrum disorder–related brain regions for a sample in the ETH dataset. sMRI: structural magnetic resonance imaging.

Those predictive features contributing most to the classification prediction of being a patient with ASD versus a healthy control were identified by calculating each cell’s feature contribution. Then, the abnormal regions identified as algorithm *high contribution features* were annotated automatically on the MRI image according to the cell’s voxel-based coordinates. Figure 7 shows the annotation of the abnormal regions of 1 specific patient with ASD from the ETH dataset. For the convenience of illustration, we annotated these regions in the form of 2D slices. In Figure 7, the number suffix of the legend on top of each slice is the slice number, and each rectangle with the red border indicates an ASD-related region.

To give a sound biological interpretation of our results, we located the standard brain regions defined in the anatomical automatic labeling (AAL) brain atlas, which is one of the most widely used cortical parcellation maps. As the AAL brain atlas is constructed on an MNI-based coordinate system, we transformed the coordinates from the voxel space into the MNI space using an affine transformation. Table 5 lists the union of ASD-related regions for all patients in the ETH dataset. The table columns *X*, *Y,* and *Z* represent the central coordinates of the disease-related cells in a 3D MNI-based space. The brain region names in the table are located based on the central coordinates. Owing to the unique set of sulcal folds for each individual, we assigned the closest region to the cell if the cell’s center did not fall in any AAL atlas region. The same method can be applied to the other 3 datasets to identify the ASD-related brain regions relevant to each dataset, and the findings show the consistency between these datasets.

**Table 5:** Autism spectrum disorder–related anatomical automatic labeling brain regions identified by a histogram-based morphometry framework on the ETH dataset.

Index	Region name	Central Montreal Neurological Institute–based coordinates^a^			Studies	
		*X*	*Y*	*Z*	Guo et al [8]	Huang et al [10]
1	Frontal_Inf_Tri_R	50	22	4	Y	N
2	Temporal_Sup_R	38	−38	4	N	N
3	Calcarine_R	32	−68	4	N	Y
4	Postcentral_R	28	−38	34	N	Y
5	Frontal_Mid_R	26	22	34	Y	Y
6	Caudate_R	20	−8	34	N	N
7	Precuneus_R	16	−38	4	N	Y
8	Caudate_R	16	22	4	N	N
9	Precuneus_L	−2	−68	34	N	Y
10	Cingulum_Mid_R	−6	22	34	Y	N
11	Precuneus_L	−8	−38	4	N	Y
12	Cingulum_Mid_L	−8	22	34	Y	N
13	Cingulum_Mid_L	−14	−38	34	Y	N
14	Precuneus_L	−18	−68	34	N	Y
15	Frontal_Sup_L	−20	52	4	Y	Y
16	Postcentral_L	−42	−8	34	N	Y
17	Temporal_Mid_L	−48	−38	4	N	N
18	Postcentral_L	−50	−8	34	N	Y
19	Lingual_R	18	−68	4	N	N
20	Insula_R	46	−8	4	Y	Y
21	Cingulum_Ant_L	−2	52	4	Y	Y
22	Pallidum_R	26	−8	4	N	N
23	Frontal_Sup_Medial_R	8	52	4	Y	Y
24	Occipital_Mid_R	−32	−68	34	N	Y
25	Parietal_Inf_L	−36	−38	34	N	N
26	Temporal_Sup_L	−50	−8	4	N	N
27	Lingual_L	−12	−68	4	N	N
28	Hippocampus_L	−24	−38	4	N	N
29	Temporal_Mid_R	−46	−38	4	N	N
30	Hippocampus_R	28	−38	4	N	N
31	Cingulum_Ant_R	16	22	34	Y	Y

^a^*X*, *Y*, and *Z* represent the central Montreal Neurological Institute–based coordinates of each disease-related cell that is located in the closest anatomical automatic labeling region. The last 2 columns represent the overlapping brain regions between our study and 2 functional magnetic resonance imaging (fMRI)–based studies (*Y* means a brain region overlaps with the fMRI-based study, whereas *N* means the opposite).

## Discussion

### Principal Findings

In this study, we developed an innovative 2-level HBM classification framework for distinguishing patients with ASD from healthy controls based on sMRI data and the 3D HOG feature extraction method. Of note, many of the brain regions utilized in our algorithm to indicate ASD—such as frontal gyrus, temporal gyrus, cingulate gyrus, postcentral gyrus, precuneus, caudate, and hippocampus—have been implicated in autism in prior neuroimaging literature [8,58-63]. Currently, ASD is a behaviorally defined disorder, diagnosed through careful clinical assessment. Our intention is not to replace the diagnostic criteria but to begin developing more objective tools which may someday augment the current ASD diagnostic process. At this juncture, we provide a proof of principle that it may be possible to develop an ASD computer-aided tool based on sMRI images alone by utilizing machine learning techniques. Of note, these techniques offer novel ways to examine neuroimaging data to probe additional clues regarding the neural underpinnings of the disorder.

Although machine learning techniques have been used in prior ASD neuroimaging studies, it is striking that most of these previous studies used fMRI rather than sMRI approaches. Our sMRI approach may represent a significant advancement given that the high cost and lower availability of fMRI likely limits its clinical applicability, while developing clinical approaches to ASD diagnosis that incorporate sMRI may be more practical given sMRI’s smaller data requirements, lower cost, and higher clinical availability. Furthermore, given that fMRI evaluates brain activation by measuring cerebral blood flow, typically during the completion of informative tasks, it is often not amenable to use for individuals with ASD. Patients being evaluated for ASD are particularly likely to have difficulty adhering to directions to complete tasks and remain still during fMRI given that they are usually children and have cognitive and/or behavioral impairments that have prompted the diagnostic evaluation. On the contrary, these concerns are well-addressed by the well-developed sedation protocols available for sMRI. In this project, using the more cost-effective sMRI approach, our ASD classification results (32/37, 86% accuracy for the ETH site) were comparable to more expensive and cumbersome fMRI approaches. For example, 2 fMRI studies based on the ABIDE I datasets have been conducted: Huang et al [10] achieved an ASD classification accuracy of 79%, while the fMRI study from Guo et al [8] obtained a classification accuracy of 86%. It should be noted that these 2 studies also used 1 site.

Of note, using our sMRI approach, we identified ASD-related brain regions that overlap with brain regions pinpointed in the above 2 fMRI studies. For example, Guo et al [8] detected ASD-associated brain function connectivities in regions, such as the inferior and superior frontal cortex, temporal cortex, cingulate cortex, and insula, which were also found to be associated with ASD in our study. Similar to Huang et al [10], we also implicated the middle frontal gyrus, middle occipital gyrus, superior frontal gyrus, calcarine cortex, and insula in ASD. The last 2 columns of Table 5 show the overlapping brain regions between our method and the above 2 fMRI-based studies. In the table cell, *Y* means a brain area identified by our method that is also reported in the studies by Guo et al [8] and Huang et al [10] and *N* means the opposite. These brain regions found to be associated with ASD by our study have striking functional correlates with the autism spectrum phenotype. Specifically, regions such as the superior temporal cortex, inferior frontal cortex, several regions of the cingulum, and the insula have been linked to social cognition and language [64]. Variations in the superior temporal gyrus have been linked to ASD-related deficits in the theory of mind (the ability to attribute mental states, such as desires and beliefs, to the self and others [65]) and face processing [66]. The inferior frontal gyrus has been associated with social functioning (including processing of facial expressions [67]) and language processing [68]. The anterior cingulate cortex has been implicated in ASD-related social impairment and repetitive behaviors [68], while the insula is involved in affective and empathic processes [69].

### Strengths and Limitations

In addition, our work represents advances over previous sMRI-based ASD neuroimaging studies, as those approaches have typically been limited by the extracted morphometry measures, such as cortical surface area and cortical thickness [16]. Importantly, these sMRI approaches are often unable to probe subcortical features, such as the amygdala and basal ganglia, which have demonstrated importance in ASD and other brain-based disorders such as Parkinson disease and depression. Our approach is amenable to the full breadth of brain structures implicated in ASD and can be easily adapted for use in other brain-based disorders. Indeed, the sMRI-based machine learning algorithm methods described herein can be adapted to study any brain disease provided that enough training data are available.

To our knowledge, this study was the first to apply a 2-level classification framework based on the 3D HOG feature extraction method to distinguish patients with ASD from healthy controls. We did not rely on 2D HOG as the layer-by-layer slicing method needed can dramatically increase training time and can lead to reduced classification accuracy owing to the separation of the image gradient information from adjacent slices. Of note, in this study we compared 3D to 2D HOG and found that 3D HOG had higher classification accuracy, as demonstrated in Figure 6. Other papers have discussed using the 3D HOG in the medical image domain [70,71]: although the 3D HOG approach may be similar to our approach, we did not concatenate the local HOG features to form a vector representing the entire image. In our framework, we extracted the 3D HOG features for local brain regions and analyzed them individually. In the first-level classification stage, we converted these local features into high-level features with the classification of diseased versus healthy, and then combined these high-level features into a vector. This means the feature dimension input to the final classifier can be considerably reduced, which helps to prevent overfitting. On the contrary, the individual local HOG features can be analyzed further to obtain their respective feature contributions to the ASD classification. These feature contributions actually depict the possibility distribution of the ASD-related brain regions based on the training data. When classifying novel individuals, the feature contributions can be used to discern the most predictive ASD-related brain regions. Importantly, our findings (Tables 3 and 4) also demonstrate that the HOG features outperform SIFT, another widely used local feature, in ASD classification. This is likely due to the ability of the HOG features to cover the entire sMRI image, ensuring that no subtle morphological abnormalities occurring in the brain are overlooked.

In addition to the strengths discussed earlier, our study has several limitations. Specifically, our HOG feature extraction method is based on the artificial division of the brain image with a fixed cell size. The abnormal regions may be located across adjacent cells, and our proposed method considers that such features have the same contribution to the classification result, which may not entirely reflect the actual grouping complexity. In the future, the HBM framework can be improved by replacing binary classification results like 0 or 1 with fuzzy numbers between 0 and 1 that represent the degree to which the image feature should be classified as a disease-related feature.

Our use of data from 4 ABIDE II sites also presents some challenges. Compared with some other available datasets such as ABIDE I, the ABIDE II datasets and sites are more heterogeneous, which may introduce classification challenges and lead to decreased case versus control classification accuracy. We noted that both Tables 3 and 4 display obvious performance variations between different sites owing to data heterogeneity (eg, differences in scanner types, data collection protocol, demographic information, and disease evaluation). When we applied the HBM method to all the data from the 4 datasets in the 10-fold CV, the resulting classification accuracy reduced to 65% (162/250). This is a common challenge when analyzing multisite data based on neuroimaging techniques. The multisite data heterogeneity makes the classifiers learn site-specific variabilities instead of important information in data themselves. If the data heterogeneous factors are not eliminated, the model performance would not improve even if trained on more data. This is evident in 4 previous studies; the accuracy ranged from 64% to 70% when data from all sites in ABIDE I were integrated [72-75]. In addition, the 2 studies that we compared also used fewer than 4 sites. In our future studies, we will endeavor to reduce the impact of sample site heterogeneity by including scanner parameters and demographic characteristics such as age, sex, and clinical measurements in the analytic models. Another method to address this limitation is through multitask learning, which considers each site as 1 task, and learning of task-shared and task-specific features simultaneously [76,77].

### Conclusions

Although ABIDE II study site heterogeneity may have limited case classification accuracy in this study, thus weakening the predictive value of our model, this study nonetheless represents the first steps in developing a classification framework that can distinguish patients with ASD from healthy controls based on the sMRI images that probe the full range of brain regions (subcortical as well as cortical) implicated in ASD. Further development of such sMRI methods—which are more affordable and clinically available than fMRI approaches—to augment the subjective clinical information currently used in the ASD diagnostic process holds much promise, as it could in the future lead to the creation of more accurate and expeditious diagnostic methods.

## Appendix

Multimedia Appendix 1 Calculation process of 3D HOG features.

Multimedia Appendix 2 Classification performance comparison for other three datasets.

## Abbreviations

AAL: anatomical automatic labeling
ABIDE I: first edition of the Autism Brain Imaging Data Exchange
ABIDE II: second edition of the Autism Brain Imaging Data Exchange
ASD: autism spectrum disorder
AUC: area under the curve
CV: cross-validation
DBM: deformation-based morphometry
DICOM: Digital Imaging and Communications in Medicine
ETH: ETH Zürich
fMRI: functional MRI
HBM: histogram-based morphometry
HOG: histogram of oriented gradients
MNI: Montreal Neurological Institute
MRI: magnetic resonance imaging
OHSU: Oregon Health and Science University
ROI: region of interest
SBM: surface-based morphometry
SIFT: scale-invariant feature transform
sMRI: structural MRI
SU: Stanford University
SVM: support vector machine
TBM: tensor-based morphometry
VBM: voxel-based morphometry
