# Supplementary material for: The Development of a Practical Artificial Intelligence Tool for Diagnosing and Evaluating Autism Spectrum Disorder: Multicenter Study
Source: JMIR Med Inform. 2020 May 8;8(5):e15767. doi: 10.2196/15767 (PMC7244998; doi:10.2196/15767)
Supplement: Multimedia Appendix 1 [file medinform_v8i5e15767_app1.docx]

Multimedia Appendix 1: Calculation process of 3D HOG features

In the process of extending the concept of HOG from 2D space to 3D space, we needed to define methods for calculating the image gradient (including direction and magnitude) and partitioning the gradient directions into a few orientation bins (or channels) in 3D space. The gradient directions in 3D space were represented by two angles θ and φ as shown in Figure 1.


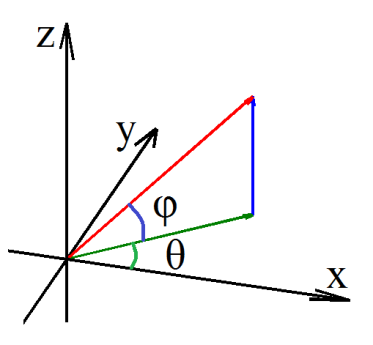


Figure 1**.** Two angles related to gradient direction calculation in 3D space.

The following equations show how to calculate the gradients of the voxels within the 3D MRI images, where the vector (Δ*I*_X_, Δ*I*_Y_, Δ*I*_Z_) is used to represent the gradient, *I* (x, y, z) is the image intensity at location (x, y, z), Δx, Δy, and Δz are small increments, which is usually set to 1 for image processing, along the three dimensions:

$\Delta I_{X}=I\left( x_{0}+\Delta x, y_{0},z_{0} \right)-I\left( x_{0}-\Delta x, y_{0},z_{0} \right)$ (1)

$\Delta I_{Y}=I\left( x_{0},y_{0}+\Delta y,z_{0} \right)-I\left( x_{0},y_{0}-\Delta y,z_{0} \right)$ (2)

$\Delta I_{Z}=I\left( x_{0},y_{0},z_{0}+\Delta z \right)-I\left( x_{0},y_{0},z_{0}-\Delta z \right)$ (3)

Based on the gradient (Δ*I*_X_, Δ*I*_Y_, Δ*I*_Z_), the two angles θ and φ representing gradient directions are calculated as follows:

$\theta=arctan\frac{\Delta I_{Y}}{\Delta I_{X}}$ (4)

$\varphi=arctan\frac{\Delta I_{z}}{\sqrt{\Delta I_{X}^{2}+\Delta I_{Y}^{2}}}$ (5)

The magnitude of the gradient is calculated as the vote weight in the orientation binning process. The equation to calculate the gradient magnitude is as follows:

$\left| \left( \Delta I_{X},\Delta I_{Y},\Delta I_{Z} \right) \right|=\sqrt{\Delta I_{X}^{2}+\Delta I_{Y}^{2}+\Delta I_{Z}^{2}}$ (6)
