# Supplementary material for: The Development of a Practical Artificial Intelligence Tool for Diagnosing and Evaluating Autism Spectrum Disorder: Multicenter Study
Source: JMIR Med Inform. 2020 May 8;8(5):e15767. doi: 10.2196/15767 (PMC7244998; doi:10.2196/15767)
Supplement: Multimedia Appendix 2 [file medinform_v8i5e15767_app2.docx]

Multimedia Appendix 2: Classification performance comparison for other three datasets

This appendix provides the following figures about classification performance comparison results of the other three datasets — ETH, OHSU, and SU.

- Figures S1, S2, and S3 are results between different HBM instances.
- Figures S4, S5, and S6 are results between 3D HOG and 2D HOG.

**Figure S1.** Classification accuracies for ETH dataset using (a) KNS26 (b) KNS32 (c) HSS26 (d) HSS32

(a)

(b)

(c)

(d)

**Figure S2.** Classification accuracies for OHSU dataset using (a) KNS26 (b) KNS32 (c) HSS26 (d) HSS32

(a)

(b)

(c)

(d)

**Figure S3.** Classification accuracies for SU dataset using (a) KNS26 (b) KNS32 (c) HSS26 (d) HSS32

(a)

(b)

(c)

(d)

**Figure S4.** Classification accuracies for ETH dataset using (a) 3D HOG (b) 2D HOG

(a)

(b)

**Figure S5.** Classification accuracies for OHSU dataset using (a) 3D HOG (b) 2D HOG

(a)

(b)

**Figure S6.** Classification accuracies for SU dataset using (a) 3D HOG (b) 2D HOG

(a)

(b)
